# Presence of periodontal pathogenic bacteria in blood of patients with coronary artery disease

**DOI:** 10.1038/s41598-022-05337-1

**Published:** 2022-01-24

**Authors:** Zuray Corredor, Andrés Suarez-Molina, Cristian Fong, Laura Cifuentes-C, Sandra Guauque-Olarte

**Affiliations:** 1 Faculty of Dentistry, Universidad Cooperativa de Colombia Campus Pasto, Pasto, Colombia; 2 Instituto Departamental de Salud de Nariño, Pasto, Colombia; 3 Faculty of Medicine, Universidad Cooperativa de Colombia Campus Santa Marta, Santa Marta, Colombia; 4 GIOM Group, Faculty of Dentistry, Universidad Cooperativa de Colombia Campus Envigado, Cra. 47 No. 37 sur 18, Envigado, Antioquia Colombia

**Keywords:** Cardiovascular diseases, Dental diseases, Metagenomics

## Abstract

It has been hypothesised that oral bacteria can migrate, through the blood, from the mouth to the arterial plaques, thus exacerbating atherosclerosis. This study compared bacteria present in the peripheral blood of individuals with and without coronary artery disease (CAD). RNA sequences obtained from blood were downloaded from GEO (GSE58150). Eight patients with coronary artery calcification (CAC) scoring > 500 and eight healthy individuals were analysed. After conducting quality control, the sequences were aligned to the hg38 reference genome using Hisat2. Bacterial taxa were analysed by inputting the unmapped sequences into Kraken. Ecological indices were calculated using Vegan. The package DESeq2 was used to compare the counts of bacteria per standard rank between groups. A total of 51 species were found only in patients with CAD and 41 were exclusively present in healthy individuals. The counts of one phylum, one class, three orders, two families and one genus were significantly different between the analysed groups (p < 0.00032, FDR < 10%), including the orders Cardiobacteriales, Corynebacteriales and Fusobacteriales. Twenty-three bacterial species belonging to the subgingival plaque bacterial complexes were also identified in the blood of individuals from both the groups; *Fusobacterium nucleatum* was significantly less frequent in patients with CAD (p = 0.0012, FDR = 4.8%). Furthermore, the frequency of another 11 bacteria differed significantly among patients with CAD than that among healthy individuals (p < 0.0030, FDR < 10%). These bacteria have not been previously reported in patients with atherosclerosis and periodontitis. The presence of members of the subgingival plaque bacterial complexes in the blood of patients with CAC supports the hypothesis that the periodontopathogens can be disseminated through the blood flow to other body parts where they may enhance inflammatory processes that can lead to the development or exacerbation of atherosclerosis.

## Introduction

Oral infections such as apical periodontitis and caries have been associated with the presence of cardiovascular diseases^1^ and with other systemic diseases such as diabetes^2^. Periodontitis is an inflammatory disease and is caused by bacteria found in the dental biofilm, which destroys the tissue surrounding the teeth, connective tissue attachment loss and alveolar bone and tooth losses^3^. According to the World Health Organisation, severe periodontitis affects 5–20% of adults globally^4^. Severe periodontitis is the sixth most prevalent disease worldwide^5^. Global population growth accounted for 67.9% of the increase in the number of prevalent cases of severe periodontitis in the past 20 years. In 2019, the number of globally prevalent cases of severe periodontitis was 1.1 billion, with this number being higher in less developed countries/regions^6^. Reportedly, there are 500–700 common oral species in different oral structures and tissues, and only approximately 280 of these species have been cultured and formally named. These oral microorganisms have been related to periodontitis, caries, and other oral infections. In periodontitis, the biofilm that is formed contains microorganisms orchestrated to maximise their adherence, communication and survival^7^. Plaque accumulation causes an inflammatory response in the host leading to gingivitis or periodontitis if the host is susceptible. These microorganisms associated with periodontitis include members of different species from the phyla Bacteroidetes, Firmicutes, Proteobacteria, Spirochaetes and Synergistetes^8^.

Several studies have shown that oral bacteria are also related to systemic diseases such as coronary artery disease (CAD), stroke and diabetes^9^. Atherosclerotic vascular disease is a condition affecting the heart and blood vessels and is usually associated with the build-up of atherosclerotic plaques inside the arteries^10^. Atherosclerosis is characterised by the accumulation of fatty and fibrous deposits in the arteries that induces the development of conditions such as CAD, peripheral vascular disease, myocardial infarction and stroke^11^.

The studies evaluating the association between dental infections and cardiovascular diseases have provided contradictory or inconclusive results^3^. In addition, no clear association has been established between dental infections and cardiovascular diseases as both these diseases share similar risk factors (i.e., obesity, insulin resistance, smoking and sedentarism)^3,12^. Irrespective, in 2015, Cotti and Mercuro^2^ assessed 20 studies published between 1989 and 2012 and established a positive correlation between oral health and cardiovascular diseases. In their review, they concluded that CAD was more prevalent in patients who underwent dental extractions owing to the presence of dental infections. Furthermore, at least four meta-analyses reviewing prospective cohort and case control studies have associated periodontitis with atherosclerotic vascular diseases, including stroke, myocardial infarction, peripheral vascular disease, abdominal aortic aneurysm, coronary heart disease and cardiovascular deaths^13–16^. A recent, national, retrospective cohort study conducted in the Taiwanese population found that males with chronic periodontitis had a significantly higher incidence risk of carotid atherosclerosis than males without chronic periodontitis^17^.

Four basic pathogenic mechanisms have been proposed to explain the relationship between oral inflammations and atherosclerosis. (1) Colonisation of arterial walls and atherosclerotic plaques by dental bacteria that enter the bloodstream. (2) Increase in systemic inflammation owing to the presence of an oral infection. Inflammatory elements released from sites of oral infections can be transported from the mouth to the atherosclerotic plaques via blood flow, thus increasing the risk of plaque rupture. (3) Activation of the host immune response to specific components of oral pathogens, thus causing autoimmunity to host proteins. (4) Pro-atherogenic effects resulting from specific bacterial toxins released by oral pathogenic bacteria^18^. In many infective and inflammatory diseases, pro-inflammatory cytokines have been attributed an important pathogenic role for promoting cell adhesion, permeability and apoptosis as part of the inflammatory response by interacting with specific receptors on various cell types. Critical events occurring during periodontal diseases have been considered a consequence of higher concentrations of different pro-inflammatory cytokines. This fact has been evidenced by the high levels of these cytokines found in diseased periodontal tissues or gingival crevicular fluid of patients with periodontitis. Likewise, persistent increases in cytokines have been associated with vascular dysfunction and vascular diseases, such as atherosclerosis and hypertension^19^.

Oxidative stress has been associated with the pathogenesis of coronary atherosclerotic complications and some of it risk factors^20^. Reactive oxygen species (ROS) and reactive nitrogen intermediates are associated with oxidative stress, which is related to multi-organ failure, endothelial damage and systemic inflammation. ROS is a primary defence factor in periodontal disease. In a periodontal inflammation model, the tissue levels of 8-OHdG were increased in the liver, heart, kidneys and brain^21^. Neutrophils, the predominant immune cells in periodontitis, produce ROS following the activation of the innate immune response to kill and remove pathogens. In 2017, Hirschfeld suggested that neutrophil stimulation with periodontal bacteria promotes extracellular, intracellular and superoxide ROS release in a possible species-specific manner^7^. Similarly, other studies have shown that periodontitis is related to excessive ROS production or elevated oxidative damage in periodontal tissue, gingival crevicular fluid or gingival blood^20^. In fibroblasts obtained from patients with periodontitis, bacterial lipopolysaccharides increased oxidative stress and mitochondrial dysfunction^22^. In these patients, elevated circulating markers of oxidative damage were observed, which was attributed to the release of ROS from the periodontal lesion into the bloodstream^22^. A lower citrate synthase activity and high levels of ROS production have also been reported in peripheral blood mononuclear cells obtained from patients with periodontitis, suggesting that this condition could lead to mitochondrial dysfunction and ROS overproduction in these cells^22^.

Metagenomics is the study of microbial communities in complex samples. The main cultivation-independent method to identify oral species is 16S rRNA gene sequencing metagenomics^9,23^. Nowadays, next-generation sequencing allows the profiling of bacterial communities in specific environments by sequencing the 16S rRNA gene^24^ or the entire genomes present in a sample^25^. Using next-generation sequencing, it is theoretically possible to identify a novel infectious specie that represents only 0.000001% of the total DNA in a clinical specimen^26^. The study by Belstrom et al*.* from 2017 is an example of the application of metagenomics in oral health; the authors found that the relative abundance of specific oral bacterial species in saliva were different among patients with caries and periodontitis^27^. However, most studies evaluating oral bacteria present in samples of patients with atherosclerosis have focused on assessing few periodontal pathogens belonging to the subgingival plaque complexes by molecular biology approaches such as polymerase chain reaction (PCR) or immunological methods. Thus, little is known about presence or the action of other oral microorganisms in the development or exacerbation of atherosclerosis in patients with periodontitis, and applying metagenomics will allow to unveil this relationship.

Several programmes have been designed to assign taxonomic labels to metagenomic DNA sequences, including MEGAN, MetaPhlAn and Kraken. The latter is an ultrafast and highly accurate programme for doing this task^28^. The reads obtained from human RNA sequencing are aligned to a human reference genome, and then the unmapped reads are processed using these computational tools to identify microorganisms present in the human sample. The NCBI GEO database^29^ contains publicly available DNA and RNA sequencing data from individuals with different phenotypes that are freely available to probe new hypothesis with respect to the original study that published the data.

In the present study, we used a set of peripheral blood samples from individuals with and without CAD available in the GEO database to compare oral microorganisms present. Overall, 41 and 51 bacterial species were identified in the healthy group and CAD group, respectively. Twenty-three bacterial species belonging to the subgingival plaque bacterial complexes were also identified in both groups, with *Fusobacterium nucleatum* being significantly less frequent in the CAD group. In addition, the frequency of another 11 bacteria differed significantly between the CAD and healthy groups, and these bacteria have not been reported before in patients with atherosclerosis and periodontitis. The role of the bacterial species identified in the present study in the initiation or exacerbation of atherosclerosis warrants investigation in future studies.

## Methods

We used RNA sequencing data available for download in the NCBI Gene Expression Omnibus (GEO) database (accession number: GSE58150). The protocols for participant examination and collection of genetic materials were approved by the National Human Genome Research Institute (NHGRI) and Boston Medical Centre Institutional Review Boards, the institutions who generated the data. All subjects who participated in the study provided informed consent for transcriptome sequencing^30^. All methods were carried out in accordance with relevant guidelines and regulations.

### Downloading public data

To compare the bacteria present in peripheral blood of individuals with and without CAD, RNA sequencing data obtained from the peripheral blood of male patients with and without coronary artery calcification (CAC) was downloaded from the NCBI GEO database^29^ (accession number: GSE58150). CAC score was obtained using multislice computed tomography. In the CAD group the CAC score was higher than 500, whereas the healthy group had a CAC score of zero (Table S1). A CAC score > 300 is considered as a predictor of future cardiovascular events. All subjects who participated in the study provided informed consent for transcriptome sequencing. More details about the characteristics of CAD and healthy groups and the sequencing methods have been reported previously by Sen et al*.* who generated the data^30^. RNA sequencing was performed using the Illumina Genome Analyser IIx (2 × 76 bp paired end read). The sample size was limited to the availability of data in GEO.

### Analysis of RNA sequences, richness and diversity

**Figure 1:**
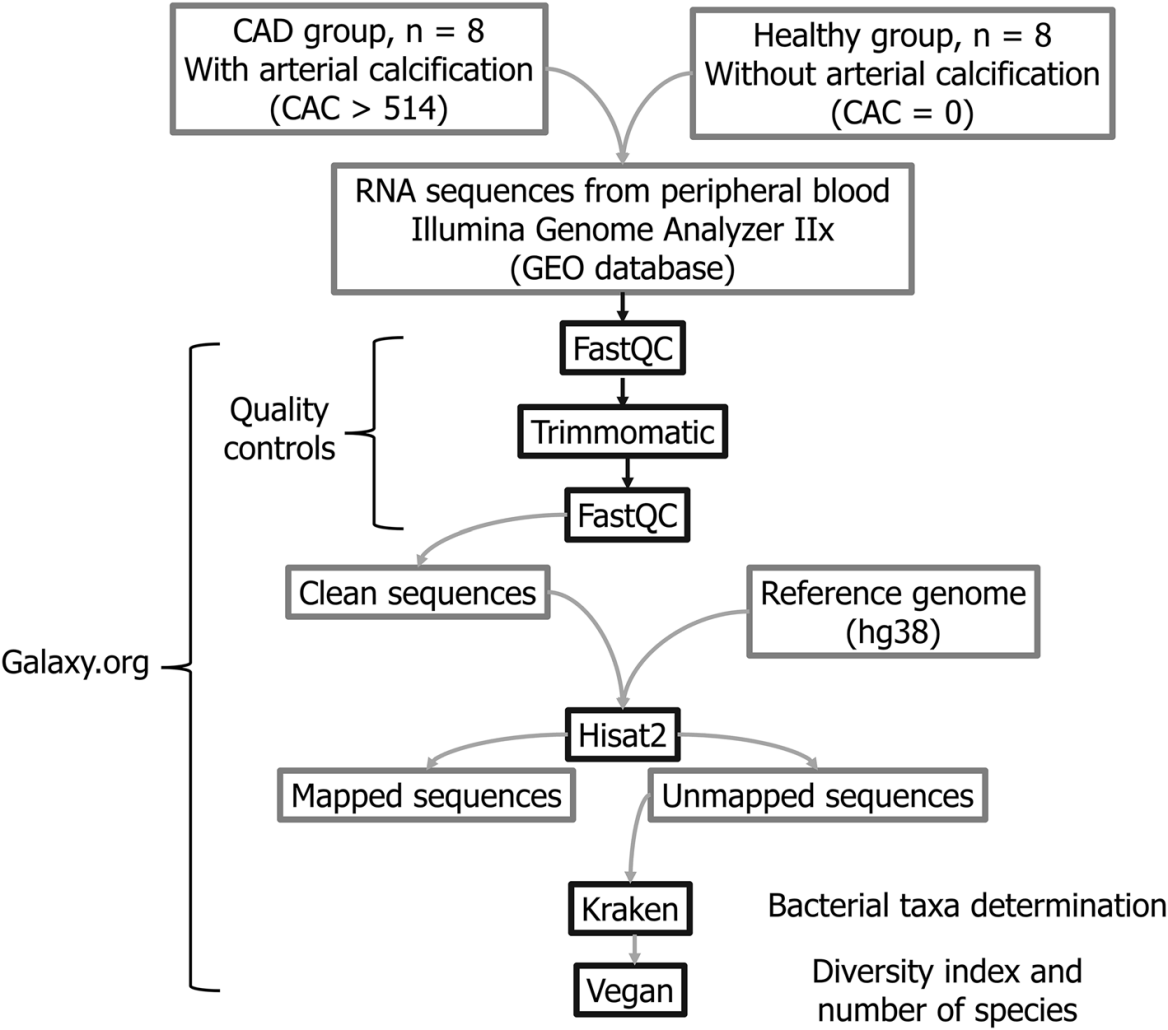
Workflow of data analysis. RNA sequences were mapped to the GRCh38 human reference genome and unmapped reads were fed into Kraken to classify the reads to bacterial taxa. The richness and diversity indices were obtained using Vegan.

Fastq files were uploaded to the Galaxy web platform, and the public server at usegalaxy.org was used to perform the analysis^31^. The quality of fastq files was evaluated using fastqc^32^ v.0.11.5. Quality control was performed using trimmomatic^33^ v.0.36. Sequences were mapped using Hisat2^34,35^ v.2.1.0 and the GRCh38 reference genome. The unmapped sequences were fed into Kraken^28^ v.1.020 to assign bacterial taxonomic label to the reads. Results of Kraken analysis were uploaded to Pavian^36^ to generate a classification report. Richness and diversity indices were calculated using Vegan^37^ v.2.5.6. Figure 1 summarises the design of the study.

### Comparison of taxa between CAD and healthy groups

The mean count of species in the CAD and healthy groups was compared applying a Welch two sample *t* test using the R package ‘picante’^38^ v.1.8, at a significance level of 5%. DESeq2 v.1.28.1 package was used to compare the counts of bacteria per standard rank (phyla, class, order, family, genus and species) between the two groups. An FDR of 10% was used to control the rate of false positives. The statistical tests were performed using R^39^ v.4.0.1 with R Studio^40^ v.1.3. 959. Venn diagrams were plotted using the web-based tool InteractiVenn^41^. Publication quality images were generated using Inkscape^42^, when possible.mendeley.

### Ethics approval and consent to participate

We used RNA sequencing data available for download in the NCBI Gene Expression Omnibus (GEO) database (accession number: GSE58150). The protocols for participant examination and collection of genetic materials were approved by the National Human Genome Research Institute (NHGRI) and Boston Medical Centre Institutional Review Boards, the institutions who generated the data. All subjects who participated in the study provided informed consent for transcriptome sequencing^30^. All methods were carried out in accordance with relevant guidelines and regulations.

## Results

### Characteristics of CAD and healthy groups

Table S1 shows the characteristics of individuals in the CAD and healthy groups. The mean age was 55.6 ± 4.8 years and 55.4 ± 4.2 years in CAD and healthy groups, respectively (p = 0.91). The mean CAC scores in the CAD group were 2059 ± 1367.7 and in the healthy group was zero (Table S1).

### Classification of reads

The reads classified by Kraken ranged from 79,088 to 172,155 in the CAD group and from 72,970 to 389,575 in the healthy group. In the CAD group, the mean of classified reads was 102,519 ± 29,620; of these reads, 82.1% corresponded to bacteria. The healthy group had a mean of 158,967 ± 103,505 classified reads, of which 88.0% were assigned to bacteria (Table S2).

### Richness and diversity indices

**Figure 2:**
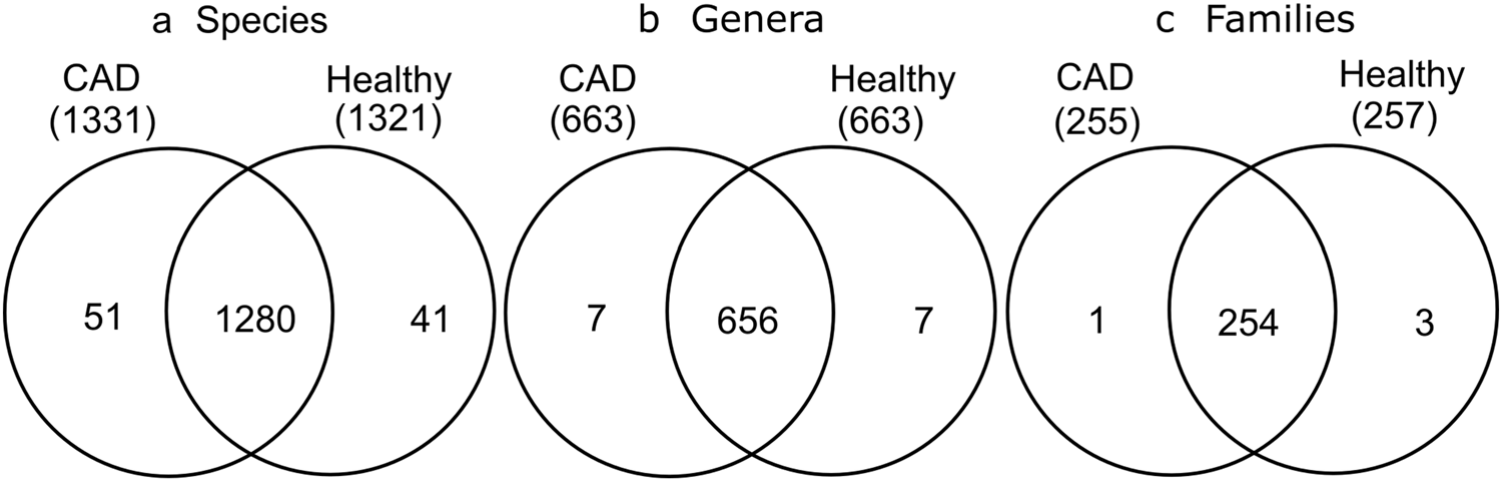
Number of (**a**) species, (**b**) genera and (**c**) families common to both CAD and healthy groups. The healthy group has a major amount of each of this taxon.

The total number of species (richness) identified in all patients with CAD was 1331 and all healthy individuals was 1321. The abundance at the level of species was 5157 in the CAD group and 4618 in the healthy group. A total of 51 species were found only in individuals with CAD and 41 were present only in healthy individuals (Fig. 2; Table 1). In the CAD group, 663 genera, 255 families, 141 orders, 60 classes and 36 phyla were detected. In contrast, in the healthy group, 663 genera, 257 families, 142 orders, 60 classes and 36 phyla were detected (Fig. 2).

**Table 1:** Most frequent species unique to coronary artery disease (CAD) and healthy groups.

Species unique to CAD group	# counts	Species unique to healthy group	# counts
*Anabaena variabilis*	4	*Candidatus Sulfuricurvum sp. RIFRC-1*^§^*	3
*Prosthecochloris aestuarii*	4	*Geobacillus thermoleovorans*^*#*^	3
*Thermodesulfatator indicus*	4	*Haloferax mediterranei*	3
Exiguobacterium sibiricum*	3	Shewanella sp. MR-4*	3
*Fervidobacterium nodosum*	3	*Thermoanaerobacter italicus*	3
*Pelodictyon phaeoclathratiforme*	3	*Candidatus Nitrosopumilus sp. AR2*^§^*	2
*Borrelia hermsii*	2	Chlamydia trachomatis*	2
Brucella ceti*	2	*Geobacillus kaustophilus*^*#*^	2
Clostridium sp. BNL1100*^#&^	2	*Phaeobacter inhibens*	2
*Mycobacterium sp. MOTT36Y*^§^*^&^	2	Shewanella halifaxensis*	2
*Natronomonas moolapensis*	2	*Thermosipho africanus*	2
*Rickettsia montanensis*	2		
Shewanella putrefaciens*	2		
*Sulfolobus tokodaii*	2		
*Thermosynechococcus elongatus*	2		
*Vibrio alginolyticus*^*†#&*^	2		
*Wolbachia endosymbiont of Brugia malayi*	2		

A total of 51 species were found only in individuals with CAD and 41 were present only in healthy individuals. The complete list of microorganisms found only in the CAD group or the healthy group is available in Table S3.

*Contaminant; ^#^Hard palate; ^&^Soft palate; ^%^Tonsils; ^†^Subgingival and supragingival plaques; ^§^Subgingival plaqu; ^¶^The Human Oral Microbiome.

**Figure 3:**
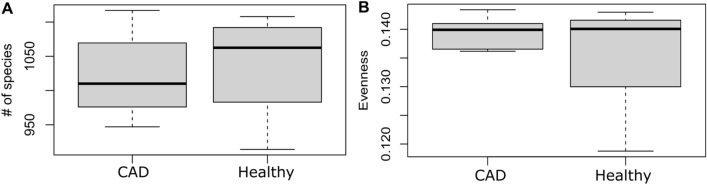
(**A**) Box plot of the mean number of species in the CAD and healthy groups. (**B**) Comparison of Simpson evenness between the CAD and healthy groups.

The mean number of species in CAD and healthy groups was 1021.9 and 1037.1, respectively (p value = 0.654) (Fig. 3A). Simpson evenness was more dispersed in the healthy group than in CAD group, albeit without significant differences (p = 0.243) (Fig. 3B). The Bray–Curtis similarity index was 0.96 (p = 0.255). Shannon and Simpson indices of diversity were not significantly different between the groups but were less dispersed in the CAD group.

### Comparison of taxa between CAD and healthy groups

DESeq2 was used to compare the counts of bacteria per phyla, class, order, family, genus and species between the CAD and healthy groups. The counts of one phylum, one class, three orders, two families and one genus were significantly different between the groups (Table 2).

**Table 2:** Significantly different taxa between the CAD and healthy groups.

Taxon	Name	log2FoldChange	p value	FDR
Phyla	Fusobacteria*^†#&^	− 2.47	0.00025	0.00914
Class	Fusobacteriia	− 2.47	0.00019	0.01137
Order	Corynebacteriales	− 3.74	0.00001	0.00091
	Fusobacteriales^#&^	− 2.38	0.00030	0.01442
	Cardiobacteriales^#&^	− 2.82	0.00031	0.01442
Family	*Corynebacteriaceae*^*#&*^	− 4.04	0.00001	0.00183
	*Cardiobacteriaceae*^*#&*^	− 2.84	0.00025	0.03212
Genus	Corynebacterium*^†$^	− 4.16	0.00001	0.00397

**Figure 4:**
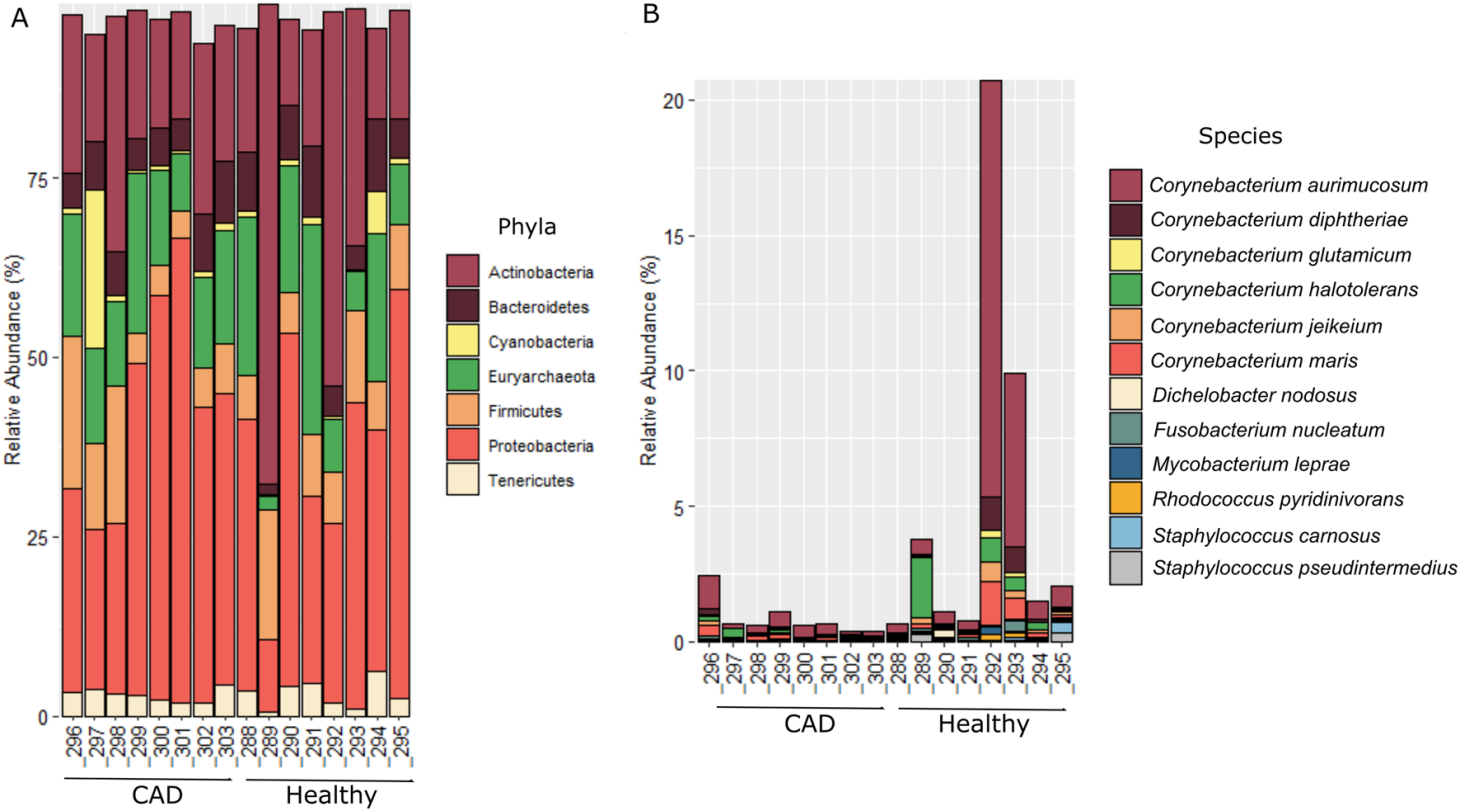
Taxonomic composition and relative abundance of the bacteria in CAD and healthy groups. (**A**) The most abundant phylum identified in the study, and (**B**) the 12 species differing significantly between the CAD and healthy groups.

The counts of 12 species differed significantly between the CAD and healthy groups, and all these species had fewer counts in the CAD group than in the healthy group (Table 3; Fig. 4). One of the significant species was *F. nucleatum* (Log2 Fold change =  − 2.63, FDR = 4.8%), a bacterium belonging to the orange subgingival plaque complex.

*Contaminant; ^#^Hard palate; ^&^Soft palate; ^%^Tonsils; ^$^Tooth surfaces; ^†^Subgingival and supragingival plaques; ^§^Subgingival.

**Table 3:** The 12 significant species between the CAD and healthy groups (FDR < 10%).

Species	log2FoldChange	p value	FDR
Staphylococcus pseudintermedius*^†#&^	− 3.51	0.0001	0.0213
Corynebacterium halotolerans*^†#&$^	− 3.81	0.0002	0.0213
Staphylococcus carnosus*^†#&^	− 3.22	0.0002	0.0213
*Rhodococcus pyridinivorans*^*&*^	− 2.65	0.0002	0.0213
*Corynebacterium diphtheriae*^*†#&$*^	− 3.12	0.0003	0.0224
*Corynebacterium glutamicum*^*†#&$*^	− 2.64	0.0006	0.0379
*Corynebacterium jeikeium*^*†#&$*^	− 2.63	0.0007	0.0379
*Dichelobacter nodosus*	− 2.72	0.0008	0.0399
Mycobacterium leprae*^†&^	− 2.55	0.0010	0.0461
Fusobacterium nucleatum*^†^*^*^*#&$%*^	− 2.63	0.0012	0.0483
Corynebacterium aurimucosum*^†#&$^	− 2.35	0.0026	0.0926
Corynebacterium maris*^†#&$^	− 2.46	0.0029	0.0954

*Contaminant; ^#^Hard palate; ^&^Soft palate; ^%^Tonsils; ^$^Tooth surfaces; ^†^Subgingival and supragingival plaques; ^§^Subgingival.

Figure 4 shows how the counts (relative abundance) of the most abundant phyla and the 12 significant species are distributed between the CAD and healthy groups. A variation in the distribution of counts among samples of the same group can be observed at the phylum and species levels; this distribution in the CAD group is more homogenous than in the healthy group. The phyla Firmicutes, Proteobacteria, Actinobacteria and Bacteroidetes were among the most frequent bacteria present in blood of patients in both the groups.

### Comparison of bacteria from periodontal bacterial complexes between CAD and healthy groups

In this study it was possible to identify oral bacteria taxa from RNA sequences derived from peripheral blood of individuals with and without CAD. Twenty-three periodontal pathogens belonging to the subgingival plaque bacterial complexes as described by Socransky and Haffajee^43,44^ were identified in peripheral blood of the CAD patients and the healthy individuals including *Aggregatibacter actinomycetemcomitans, Tannerella forsythia, Prevotella intermedia, Prevotella denticola, Treponema denticola* and *Porphyromonas gingivalis* (Table 4). The bacterial counts of *Fusobacterium nucleatum* was significantly different between the CAD and healthy groups (Log2 Fold change =  − 2.63, p = 0.0012, FDR = 4.8%). *Porphyromonas gingivalis* was significantly more frequent in the CAD group before adjustment for multiple testing (p = 0.030).

**Table 4:** Subgingival plaque bacterial complexes and their species as described by Socransky and Haffajee.

Specie	Complex	Specie	Complex
*Actinomyces species*	A	*Fusobacterium periodonticum*	O
Veillonella parvula*	P	*Parvimonas micra*	O
*Actinomyces odontolyticus*	P	Prevotella intermedia*	O
Streptococcus sp*	Y	*Prevotella nigrescens*	O
Streptococcus gordonii*	Y	Streptococcus constellatus*	O
Streptococcus intermedius*	Y	*Eubacterium nodatum*	O
Streptococcus mitis*	Y	Porphyromonas gingivalis*	R
Streptococcus oralis*	Y	Tannerella forsythia*	R
Streptococcus sanguinis*	Y	Treponema denticola*	R
*Capnocytophaga gingivalis*	G	Aggregatibacter actinomycetemcomitans*	U
Capnocytophaga ochracea*	G	*Selenomonas noxia*	U
*Capnocytophaga sputigena*	G	*Corynebacterium matruchotii*	Other
*Capnocytophaga concisus*	G	*Eubacterium saburreum*	Other
*Eikenella corrodens*	G	*Eubacterium sulci*	Other
Aggregatibacter actinomycetemcomitans a*	G	*Gemella morbillorum*	Other
*Campylobacter gracilis*	O	Leptotrichia buccalis*	Other
*Campylobacter rectus*	O	*Porphyromonas endodontalis*	Other
*Campylobacter showae*	O	Prevotella melaninogenica*	Other
Fusobacterium nucleatum*	O	Propionibacterium acnes*	Other
Fusobacterium nucleatum vicentii*	O	*Neisseria mucosa*	Other
Fusobacterium nucleatum nucleatum*	O	Streptococcus anginosus*	Other
Fusobacterium nucleatum polymorphum*	O	*Treponema socranskii*	Other

The species identified in the present study are marked with an asterisk. The proportion of counts of *Fusobacterium nucleatum* was significantly less frequent in the CAD patients compared to the healthy individuals. *Porphyromonas gingivalis* was significantly more frequent in the CAD group before adjustment for multiple testing.

Aggregatibacter actinomycetemcomitans, Fusobacterium nucleatum subspecies were aggregated in one group according to their specie. Complex: A, Actinomyces; P, Purple; Y, Yellow; G, Green; O, Orange; R, Red; U, Ungrouped.

## Discussion

The present study identified periodontopathogens in the peripheral blood of patients with and without CAD using RNA sequences obtained from a public database. In total, 51 species were found only in the CAD group and 41 were found exclusively in the healthy group. Thus, 3.8% of species identified were not common between the CAD and healthy groups, which means that the habitat (blood from the two groups) can explain taxonomic, phylogenetic and trait dissimilarities among the individuals studied. The most frequent bacterial phyla identified by the Human Microbiome Project Consortium in healthy adults (Firmicutes, Proteobacteria, Actinobacteria, Bacteroidetes and Fusobacteria) were also among the most frequent bacteria identified in the bloodstream of CAD patients and healthy individuals in the present study (Fig. 4). Although the counts were normalised based on the total number of reads per sample, the distribution of counts varied among samples of the same group; however, at the level of species, count distribution in the CAD group was more homogenous.

Interindividual variation in the host microbiota composition has been observed for oral communities in both healthy and diseased states because of differences in the environment, genetics, age and lifestyle. As core functions of the oral microbiota can be accomplished by different groups of bacteria, this heterogeneity is prone to occur^9,45^. In addition, if different factors influence the infiltration of oral bacteria into the bloodstream, the blood bacterial composition in different individuals may differ too. Hyperlipidemia is considered a major risk factor for coronary heart disease^46^ and statins are the most widely used treatment for hyperlipidemia^47^. Statins can modify oral^48^ and gut microbiota (community diversity and taxon abundance)^46,47^. Kamińska et al*.* performed an in vitro study to assess the antimicrobial properties of fluvastatin, atorvastatin, lovastatin and simvastatin against oral bacteria associated with periodontitis (*Porphyromonas gingivalis*, *Fusobacterium nucleatum*, *Actinomyces naeslundii*, *Tannerella forsythia*, *Streptococcus gordonii*). They observed a statin dose-dependent effect to induce significant antimicrobial activities above the plasma concentration reached by atorvastatin or simvastatin when administered systemically. In our study, we do not have access to patients’ statin or other drugs prescriptions that are usually taken by CAD patients but it would be possible that the blood microbiota in the CAD group may be influenced by those drugs but even though, bacteria were detected in their blood.

We did not observe significant differences in bacterial diversity between the CAD and healthy groups but there were some unique species identified only in either groups. Among the unique species identified in patients with CAD, the *Mycobacterium *sp. MOTT36Y was previously reported in subgingival plaque^9^; *Clostridium *sp. BNL1100 have been found in hard and soft palates and *Vibrio alginolyticus* was previously found in hard and soft palate, subgingival and supragingival plaques^9,49^. In contrast, the unique species present in the healthy group have been previously identified in subgingival plaque (*Candidatus Sulfuricurvum *sp. RIFRC-1, *Candidatus Nitrosopumilus *sp. AR2, *Bartonella tribocorum*, *Campylobacter coli*^49^, *Candidatus Blochmannia pennsylvanicus* and *Candidatus Methanomethylophilus alvus*) and hard and soft palates (*Geobacillus thermoleovorans*, *Geobacillus kaustophilus*, *Vibrio alginolyticus* and *Campylobacter coli*)^9,49^. Additionally, in the healthy group, we identified bacteria that were present in other mouth tissues of healthy individuals such as *Acidilobus saccharovorans*^9^ and *Borrelia miyamotoi*^49^.

A significantly lower count of bacteria was found in the blood of the CAD group than in that of the healthy group; this was true for the phylum Fusobacteria; the class Fusobacteriia; the orders Corynebacteriales, Fusobacteriales and Cardiobacteriales; the families *Corynebacteriaceae* and *Cardiobacteriaceae* and the genus *Corynebacterium* (Table 2). Members of the phylum Fusobacteria have been found in dental plaque on the teeth or subgingival periodontal pockets as well as hard and soft palates. Members of the order Fusobacteriales were previously identified in hard and soft palates^9^. A study focused on the analysis of oral microbial communities of individuals with oral health found that the predominant taxon was the phylum Fusobacteria (genus *Fusobacterium*)^50^. This phylum was also the dominant taxa in healthy plaque microbiota^51^. Despite the latter, the phylum Fusobacteria is a common obligately anaerobic gram-negative bacteria in the oral cavity that may act as a bridge between early and late colonising bacteria in dental plaque and has a role in oral and extra-oral infections^52^. This phylum was dominantly present in patients with periodontitis^53^, but articles focusing on subgingival microbial diversity have shown that the diversity of Fusobacteria was significantly lower in patients ﻿with chronic periodontitis^54^. In a microbiome analysis of the oral cavity among patients with internal carotid artery stenosis, Isoshima et al*.* found that *Fusobacterium* was more frequent in patients with periodontitis than in those without this oral disease^55^.

In the present study, the proportion of the order Cardiobacteriales, the families *Corynebacteriaceae* and *Cardiobacteriaceae* and the genus *Corynebacterium* was significantly lower in the blood of patients with CAD. These bacterial families have been previously identified in the hard and soft palates, whereas the genus has been identified in the dental plaque of teeth or subgingival periodontal pockets, hard and soft palates and dental surfaces^49^. Different genera of the *Cardiobacterium* family are a cause of endocarditis and infections of the heart valves and have been attributed to be the cause of other infections, such as bacteraemia, sepsis, septic arthritis, peritonitis, pericarditis, meningitis, osteomyelitis, eye infections, periodontal infections and abscesses^56^.

From the 12 significant species between the CAD and healthy groups (Table 3), 11 have been detected in dental plaque from the teeth or subgingival periodontal pockets, hard and soft palates (*Staphylococcus pseudintermedius, Corynebacterium halotolerans, S. carnosus, Rhodococcus pyridinivorans, C. diphtheriae, C. glutamicum, C. jeikeium, Mycobacterium leprae, Fusobacterium nucleatum, C. aurimucosum* and *C. maris*). Furthermore, the periodontal pathogen, *F. nucleatum*, has been found on the back of the tongue, on the hard and soft palates, tonsils, dental surfaces and subgingival plaque. *C. diphtheriae*, considered as non-toxigenic, can cause diseases such as endocarditis and septic arthritis in certain vulnerable populations^57^. *C. aurimucosum* is detected rarely in human clinical specimens and has been recovered from blood cultures of a patient with bronchitis^58^ and from patients with rheumatoid arthritis^59^. The genus *Corynebacterium* of the family *Corynebacteriaceae* includes 44 species isolated from humans; six significant species observed in the present study belong to this genus: *C. halotolerans, C. diphtheriae, C. glutamicum, C. jeikeium, C. aurimucosum* and *C. maris* (Table 3). *C. aurimucosum* was isolated from the blood of a patient with bronchitis. It has also been isolated from joint and bone infections. *C. jeikeium*, a strictly aerobic microorganism, is associated with infectious disease in humans, it has been isolated from blood and it is responsible for sepsis in hospitalised immunocompromised patients and endocarditis, was present in mechanical prosthetic valve^60^ and in infection of osteomyelitis^61,62^. Histopathological examinations have reported *M. leprae* on the epithelial surface of oral leprosy lesions^63^.

As periodontal disease severity increases, microbiological shift occurs from low levels of pathogenic bacteria in the subgingival plaque (‘incipient’ dysbiosis) to a higher level of gram-negative, strictly anaerobic pathogens. Patients with the severe periodontal disease show the highest subgingival colonisation of orange and red complex bacteria, and this result is consistent with a dysbiotic state^64^. In the dental plaque of individuals with periodontitis, the microbiota is diverse, and the main species found in dental plaque belong to the phyla Bacteroidetes, Firmicutes, Proteobacteria, Spirochaetes and Synergistetes^65^. We identified 23 species belonging to the subgingival plaque bacterial complexes as described by Socransky and Haffajee. *F. nucleatum* was significantly less frequent in patients with CAD than in healthy individuals (Table 3). *F. nucleatum* (phylum Fusobacteria) is a bacteria belonging to the orange complex^43,44^ and is an obligate anaerobic gram-negative fusiform bacillus^66^, capable of systemic dissemination (causing infections and abscesses) and has the ability to adhere to and invade different types of host cells^67^. The genus *Fusobacterium* was related to the pathogenicity of generalised aggressive periodontitis. *F. nucleatum* co-aggregates with early and late bacteria colonisers of the dental plaque, and it has been previously suggested that *F. nucleatum* is an important ‘bridge’ microorganism in the succession of genera in naturally developing dental plaque. The ability of *F. nucleatum* to co-aggregate is essential for the formation of multi-species biofilms^68^. Anaerobic bacteria biofilm formation leads to an inflammatory process, which later spreads to the deeper connective tissues. This inflammatory process is mediated by osteoclasts, which are primarily triggered by the pro-inflammatory molecule PGE2 and are clinically detected in periodontal pockets. This leads to the destruction of the periodontal supporting tissues and leads to alveolar bone loss^69^, unleashing periodontitis, which is the most common chronic inflammatory condition worldwide and is associated with incident CAD^70^.

A recent study explored the relationship between periodontal disease, a chronic inflammatory condition, and CAC and found that periodontal disease correlates positively and linearly with the presence of CAC^71^. A study focused on multiracial population found a significant association between the degree of periodontal disease and cardiac calcification^70^. Furthermore, higher periodontal scores were associated with greater degrees of calcification^70^. *F. nucleatum* adheres to and invades host epithelial and endothelial cells via a surface adhesin FadA. This adhesin is highly conserved in oral Fusobacteria, such as *F. nucleatum*, whereas it is not found in non-oral fusobacterial species^72^. The secreted mFadA is exposed on the bacterial surface, whereas pre-FadA is anchored in the inner membrane. Together, mFadA and pre-FadA form a high molecular-weight complex (FadAc), required for the attachment and invasion of host cells^73^. Vascular endothelial (VE)-cadherin is a member of the cadherin family and a cell–cell junction molecule; it was identified as the endothelial receptor for FadA, required for *F. nucleatum* to bind to the cells. FadA co-localised with VE-cadherin on endothelial cells, causing relocation of VE-cadherin away from the cell–cell junctions. As a result, the endothelial permeability increased, allowing the bacteria to cross the endothelium through loosened junctions. This crossing mechanism may explain why the organism can disseminate systemically to colonise different body sites and even overcome the blood–brain barrier. In vitro studies on migration and endothelial response (Transwell assays) suggest that *F. nucleatum* may serve as an ‘enabler’ for other microorganisms to spread systemically. This might explain why *F. nucleatum* is often found in mixed infections^67^. These observations could suggest that periodontal pathogens in coinfection can affect aortic endothelial cells and trigger some degree of calcification in human aortic endothelial cells^74^.

In 2017, Mougeot et al*.*^75^ compared bacterial species in tissue samples from clinically non-atherosclerotic areas of coronary and femoral arteries of 42 patients with atherosclerotic cardiovascular disease. This study provided insights into the presence and the types of bacteria found in clinically healthy arterial tissues, which may be associated with the initiation and/or exacerbation of atherosclerosis, with or without any role in causation. In our study, seven of the 10 most frequent species identified by Mougeot et al*.* were also identified in CAD patients and healthy individuals: *Porphyromonas gingivalis, Enterococcus faecalis, Finegoldia magna, Pseudomonas aeruginosa, Haemophilus parainfluenzae, Rothia mucilaginosa* and *Stenotrophomonas maltophilia*. In our study, *P. gingivalis* was significantly less frequent in the blood of CAD patients before adjustment for multiple testing (p = 0.030). In the study by Mugeout et al*.*, *P. gingivalis* was the most predominant species identified. However, they did not compare healthy tissues with atherosclerotic tissues. A possible explanation of the less frequency of *P. gingivalis* in the blood of patients with CAD in the present study could be that the disfunction of arterial endothelium in patients with CAD may facilitate the infiltration of bacteria such as *P. gingivialis* into the artery wall, thus lowering the bacteria in the bloodstream. In addition to other atherosclerosis risk factors, dysfunction of the endothelium may be mediated by *F. nucleatum*, which we also found to be significantly less frequent in the blood of patients with CAD than healthy individuals. *F. nucleatum* can bind to endothelial cells^67,73^ and can enhance invasion of aortic endothelial cells by *P. gingivalis*^74^. Coinfection with *F. nucleatum* resulted in a 2- to 20-fold increase in host cell invasion by *P. gingivalis* strains^74^.

An important part of a metagenomic research is to identify bacteria that are common contaminants in the laboratory environment or reagents. The following species that have been identified previously as contaminating microbiota in non-oral microbiota studies, *Exiguobacterium sibiricum, Brucella ceti,* and *Shewanella putrefaciens*^26,76^ were observed in the CAD group. Furthermore, the following species that have been previously identified as contaminating microbiota in non-oral studies, *Chlamydia trachomatis, Shewanella sp. MR-4* and *Shewanella halifaxensis*^26,76^ were detected in the healthy group.

The limitations of this study include the small sample size that was limited to the availability of the data in GEO. In addition, the data are mRNA sequences and not total RNA sequences, which could lead to missing some bacterial taxa that may be present in human blood samples. We do not have access to any clinical data other than the coronary artery calcification scoring and thus, we cannot address if the changes in the microbiota are causative or are modified by drugs used by patients.

## Conclusions

We compared the oral microorganisms present in peripheral blood of individuals with and without coronary artery calcification and identified differences in the frequency of some bacterial species. Some of the unique bacteria have been previously reported in hard and soft palate, subgingival and supragingival plaques. Twelve bacteria have a significantly different frequency in CAC than in a healthy state. To the best of our knowledge, these bacterial species have not been reported before in patients with atherosclerosis; however, some of them are known to be toxic and related to infectious diseases/processes. Bacteria from the subgingival plaque bacterial complexes were present in the blood of individuals from the two groups, including *F. nucleatum* which was significantly less frequent in CAC. The presence of subgingival plaque members in the blood of patients with CAC supports the hypothesis that pathogens from subgingival plaque can be disseminated from the oral cavity to the arteries through the blood flow, thus enhancing inflammatory processes that can lead to the development or exacerbation of systemic diseases such as CAD. Our study shows that the identification of bacterial pathogen in well phenotype patients with periodontitis and CAD using high-throughput sequencing methods is recommended to make a better profile of the microbial communities migrating from the oral cavity to the bloodstream and arteries. The impact of the bacterial species identified in the present study in the initiation or exacerbation of atherosclerosis warrants future investigation*.*

## Supplementary Information

Supplementary Information.

## Data Availability

The datasets analysed during the current study are available in the NCBI GEO database (accession number: GSE58150, https://www.ncbi.nlm.nih.gov/geo/query/acc.cgi?acc=GSE58150).
