# Supplementary material for: Presence of periodontal pathogenic bacteria in blood of patients with coronary artery disease
Source: Sci Rep. 2022 Jan 24;12:1241. doi: 10.1038/s41598-022-05337-1 (PMC8786953; doi:10.1038/s41598-022-05337-1)
Supplement: Supplementary file 1 — Supplementary Information. [file 41598_2022_5337_MOESM1_ESM.pdf]

Supplementary Tables

Presence of periodontal pathogenic bacteria in blood of patients with coronary artery disease

Zuray Corredor[1], Andrés Suarez-Molina[2], Cristian Fong[3], Laura Cifuentes-C[1], Sandra Guauque-Olarte[4]

[1] Faculty of Dentistry, Universidad Cooperativa de Colombia campus Pasto, Colombia

[2] Instituto Departamental de Salud de Nariño, Colombia

[3] Faculty of Medicine, Universidad Cooperativa de Colombia campus Santa Marta, Colombia

[4] Faculty of Dentistry, Universidad Cooperativa de Colombia campus Envigado, Colombia

Table S1. Age and coronary artery calcium (CAC) score of the CAD and healthy individuals. The CAC was measured by multislice computer tomography.

CAD group			Healthy group		
**ID**	**Age**	**CAC**	**ID**	**Age**	**CAC**
SRR1313301	55	3532	SRR1313293	55	0
SRR1313302	64	2885	SRR1313294	63	0
SRR1313303	57	1693	SRR1313295	56	0
SRR1313297	61	1370	SRR1313289	60	0
SRR1313296	54	1114	SRR1313288	54	0
SRR1313298	51	1012	SRR1313290	52	0
SRR1313300	53	4352	SRR1313292	52	0
SRR1313299	50	514	SRR1313291	51	0
**Mean ± SD**	**55.6 ± 4.8**	**2059 ± 1367.7**	**Mean ± SD**	**55.4 ± 4.2**	**0**

CAC: coronary artery calcification; CAD: Coronary artery disease.

Table S2. Number of total and classified reads in CAD patients and healthy individuals.

**Sample ID CAD**	**# classified reads**	**# bacterial reads**	**% bacterial reads**	**Sample ID Healthy**	**# classified reads**	**# bacterial reads**	**% bacterial reads**
SRR1313296	172155	137980	80,1	SRR1313288	72970	54334	74,5
SRR1313297	94440	78030	82,6	SRR1313289	389575	379675	97,5
SRR1313298	106623	89464	83,9	SRR1313290	104517	81106	77,6
SRR1313299	99950	74850	74,9	SRR1313291	73029	49072	67,2
SRR1313300	89035	74527	83,7	SRR1313292	126326	113514	89,9
SRR1313301	97490	86976	89,2	SRR1313293	208471	194114	93,1
SRR1313302	79088	66514	84,1	SRR1313294	135602	103813	76,6
SRR1313303	81367	64675	79,5	SRR1313295	161246	143459	89,0
**Mean**	102519	84127	82,1	**Mean**	158967	139886	88,0
**Median**	95965	76440	79,7	**Median**	130964	108664	83,0
**Std dev**	29620	23424	-	**Std dev**	103505	107882	-

Table S3. Complete list of species unique to the CAD group and the healthy group with their frequencies.

**Species unique to CAD group**	**Counts**	**Species unique to healthy group**	**Counts**
Anabaena variabilis	4	*Candidatus Sulfuricurvum sp. RIFRC-1*	3
Prosthecochloris aestuarii	4	*Geobacillus thermoleovorans*	3
Thermodesulfatator indicus	4	*Haloferax mediterranei*	3
Exiguobacterium sibiricum	3	*Shewanella sp. MR-4*	3
Fervidobacterium nodosum	3	*Thermoanaerobacter italicus*	3
Pelodictyon phaeoclathratiforme	3	*Candidatus Nitrosopumilus sp. AR2*	2
Borrelia hermsii	2	*Chlamydia trachomatis*	2
Brucella ceti	2	*Geobacillus kaustophilus*	2
Clostridium sp. BNL1100	2	*Phaeobacter inhibens*	2
Mycobacterium sp. MOTT36Y	2	*Shewanella halifaxensis*	2
Natronomonas moolapensis	2	*Thermosipho africanus*	2
Rickettsia montanensis	2	*Acidilobus saccharovorans*	1
Shewanella putrefaciens	2	*Bartonella tribocorum*	1
Sulfolobus tokodaii	2	*Borrelia miyamotoi*	1
Thermosynechococcus elongatus	2	*Campylobacter coli*	1
Vibrio alginolyticus	2	*Candidatus Blochmannia pennsylvanicus*	1
Wolbachia endosymbiont of Brugia malayi	2	*Candidatus Methanomethylophilus alvus*	1
Borrelia valaisiana	1	*Candidatus Nitrosopumilus koreensis*	1
Candidatus Blochmannia chromaiodes	1	*Candidatus Ruthia magnifica*	1
Cenarchaeum symbiosum	1	*Chlamydia muridarum*	1
Chlamydophila abortus	1	*Chlamydia pneumoniae*	1
Chlamydophila felis	1	*Dictyoglomus thermophilum*	1
Haloarcula marismortui	1	*Geobacillus sp. Y4.1MC1*	1
Halobacterium salinarum	1	*Haloarcula hispanica*	1
Halorhabdus utahensis	1	*Leuconostoc kimchii*	1
Helicobacter acinonychis	1	*Leuconostoc sp. C2*	1
Helicobacter hepaticus	1	*Listeria seeligeri*	1
Kinetoplastibacterium blastocrithidii	1	*Methanoregula formicica*	1
Lactobacillus amylovorus	1	*Methanosaeta thermophila*	1
Listeria welshimeri	1	*Methanosphaera stadtmanae*	1
Methanocaldococcus jannaschii	1	*Methanothermobacter marburgensis*	1
Methanocella arvoryzae	1	*Methanotorris igneus*	1
Methanothermobacter thermautotrophicus	1	*Mycoplasma pneumoniae*	1
Mycoplasma synoviae	1	*Pyrococcus furiosus*	1
Natrinema sp. J7-2	1	*Rickettsia conorii*	1
Natronomonas pharaonis	1	*Rickettsia japonica*	1
Pseudothermotoga thermarum	1	*Streptococcus phage YMC-2011*	1

Pyrobaculum calidifontis	1	*Thermococcus gammatolerans*	1
Pyrobaculum oguniense	1	*Thermotoga neapolitana*	1
Rickettsia felis	1	*Vibrio sp. Ex25*	1
Rickettsia massiliae	1	*Xanthomonas axonopodis*	1
Rickettsia typhi	1		
Sulfuricurvum kujiense	1		
Thermoanaerobacter pseudethanolicus	1		
Thermococcus barophilus	1		
Thermogladius cellulolyticus	1		
Thermoplasmatales archaeon BRNA1	1		
Thermosynechococcus sp. NK55a	1		
Ureaplasma parvum	1		
Vulcanisaeta distributa	1		
Wolbachia endosymbiont of Drosophila melanogaster	1		
